# Machine Learning Approach for Analyzing 3-Year Outcomes of Patients with Brain Arteriovenous Malformation (AVM) after Stereotactic Radiosurgery (SRS)

**DOI:** 10.3390/diagnostics14010022

**Published:** 2023-12-22

**Authors:** Mirko Jerber Rodríguez Mallma, Marcos Vilca-Aguilar, Luis Zuloaga-Rotta, Rubén Borja-Rosales, María Salas-Ojeda, David Mauricio

**Affiliations:** 1 Facultad de Ingeniería Industrial y de Sistemas, Universidad Nacional de Ingeniería, Lima 15333, Peru; 2 Instituto de Radiocirugía del Perú, Clínica San Pablo, Lima 15023, Peru; 3 Servicio de Neurocirugía, Hospital María Auxiliadora, Lima 15828, Peru; 4 Universidad San Ignacio de Loyola, Lima 15024, Peru; 5 Universidad Nacional Mayor de San Marcos, Lima 15081, Peru

**Keywords:** brain arteriovenous malformation, prognosis, prediction, machine learning, artificial intelligence, decision tree, logistic regression

## Abstract

A cerebral arteriovenous malformation (AVM) is a tangle of abnormal blood vessels that irregularly connects arteries and veins. Stereotactic radiosurgery (SRS) has been shown to be an effective treatment for AVM patients, but the factors associated with AVM obliteration remains a matter of debate. In this study, we aimed to develop a model that can predict whether patients with AVM will be cured 36 months after intervention by means of SRS and identify the most important predictors that explain the probability of being cured. A machine learning (ML) approach was applied using decision tree (DT) and logistic regression (LR) techniques on historical data (sociodemographic, clinical, treatment, angioarchitecture, and radiosurgery procedure) of 202 patients with AVM who underwent SRS at the Instituto de Radiocirugía del Perú (IRP) between 2005 and 2018. The LR model obtained the best results for predicting AVM cure with an accuracy of 0.92, sensitivity of 0.93, specificity of 0.89, and an area under the curve (AUC) of 0.98, which shows that ML models are suitable for predicting the prognosis of medical conditions such as AVM and can be a support tool for medical decision-making. In addition, several factors were identified that could explain whether patients with AVM would be cured at 36 months with the highest likelihood: the location of the AVM, the occupation of the patient, and the presence of hemorrhage.

## 1. Introduction

Cerebral arteriovenous malformation (AVM) is a congenital neurological disease that causes cerebral hemorrhage, seizures, or headache. It consists of an abnormal conglomerate of dilated cerebral vessels derived from the maldevelopment of the capillary network that allows direct connections between cerebral arteries and veins [1]. One of the treatments, known of since the 1970s, in addition to microsurgery and endovascular therapy, is stereotactic radiosurgery (SRS), in which the AVM is obliterated by radionecrosis through the administration of multi-beam directed radiation [2]. From the medical point of view, SRS is a neurosurgical technique that does not require an incision and is used as an alternative or complement to noninvasive treatment.

The healing process of patients with AVM undergoing SRS is not immediate and requires time with clinical and imaging monitoring to know the evolution of the disease. The successful exclusion of brain AVM with radiosurgery is considerably higher for smaller lesions. For example, one study showed that the obliteration rate of patients with brain AVM after SRS was between 54–92% for lesion diameters ≤ 2.5 cm [3]. Several scoring systems, such as the Spetzler–Martin Grading Scale (SMGS) and the Virginia Radiosurgery AVM Scale (VRAS), are currently used by physicians to understand the nature of AVM and predict the results of radiosurgery treatment [4,5,6]. However, developing new methods to predict the results of radiosurgery treatment and determining the factors that influence the probability of success are needed.

Machine learning (ML) is a subset of artificial intelligence (AI) that uses algorithms that automatically “learn” to identify patterns in data, which are used to make forecasts based on these patterns [7]. The use of such algorithms as support tools for medical decision-making and their application in the prognosis, diagnosis, and treatment of diseases has been recently developed [8]; however, certain conditions still exist that make it difficult for them to be widely adopted [9,10,11,12,13]. Among the studies referring to the prediction and diagnosis of neurological and brain diseases in which ML techniques were applied is the study of Uspenskaya-Cadoz et al. [14], which proposed a method for diagnosing Alzheimer’s disease (AD) by applying logistic regression (LR), decision tree (DT), random forest (RF), and gradient-boosted trees (GBT) techniques, and the study of Ghafouri-Fard et al. [15], which proposed using artificial neural networks (ANNs) to predict multiple sclerosis (MS) risk based on genotypes.

At present, the application of ML techniques to the diagnosis, prognosis, or treatment of AVM has increased. Interesting studies can be found, such as one by Tao et al. [16], which examined the factors that influence the risk of bleeding from AVM, and another by Hong et al. [17], which reported an experiment for the detection of hemorrhages in AVMs using digital subtraction angiography (DSA) images. There are also studies on the use of deep learning models, a type of ML specialized in image processing; for example, Wang et al. [18] automated the process of segmenting and identifying AVMs in computed tomography (CT) and DSA images. Other studies have focused on the prognosis of patients with AVM after surgery, with the aim of predicting whether they would be cured. For example, Asadi et al. [19] presented a study on identifying the factors that influence the outcome of treatment with endovascular embolization and showcased that ML techniques can satisfactorily predict outcomes with high accuracy and can help to individualize the treatment based on key predictors. Finally, Oermann et al. [20] used an ML approach to predict the outcomes of AVM patients undergoing radiosurgery, and achieved an accuracy of 0.74, which is considered to be the best prognostic result as of the date of publication of this paper. However, the prediction error rate found in these previous studies is high (greater than 25%), and in addition, they did not study the explainability phenomena through assessing the importance of the variables, which is key for medical decision-making.

From these previous studies [16,17,18,19,20], which show that ML algorithms are powerful tools that can be used in the medical field, in the present study, we aimed not only to provide an ML approach for predicting whether patients with AVM who undergo SRS will be cured but also one that could identify the main factors influencing whether these patients will be cured 36 months after radiosurgery.

## 2. Materials and Methods

**Figure 1:**
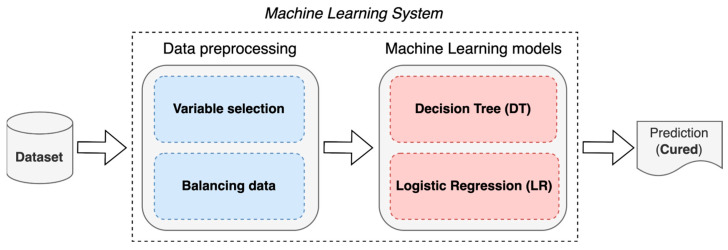
Proposed ML system.

The construction of an ML system for the prognosis of patients with AVM treated with SRS is proposed using two techniques: DT and LR (Figure 1). These two techniques were used in this study because they can produce results (predictions) that are easy to understand by the experts in the domain as they are considered “white box” methods [21]. Additionally, these methods were also used in previous studies regarding the AVM outcome prediction [16,19,20].

Due to a common long-term follow-up protocol that suggests complete AVM obliteration within the first 3 years for 70–80% of AVM patients [22], the objective of this study is to predict whether a patient will be cured or not at 36 months after undergoing SRS; for this, a supervised ML learning approach was chosen via binary classification. Additionally, the use of LR is proposed to determine the main factors that influence whether an AVM patient will be cured.

### 2.1. Dataset

**Figure 2:**
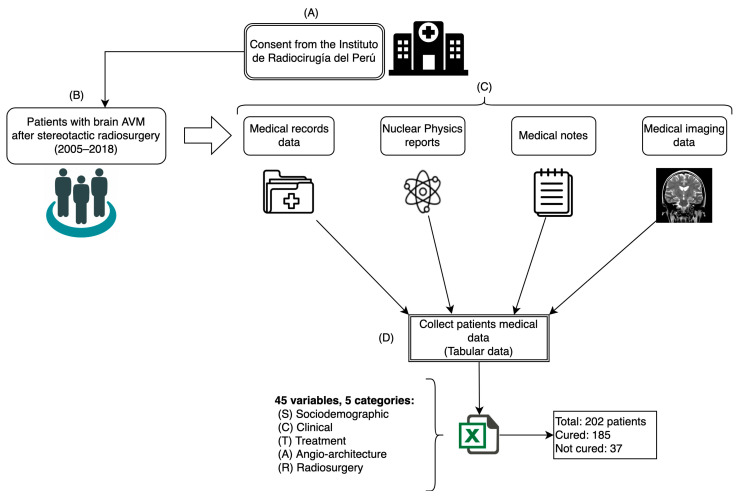
Data collection process: (**A**) consent; (**B**) patient selection; (**C**) data extraction; (**D**) data tabulation.

For this study, a dataset comprising 45 variables of 202 patients diagnosed with AVM who underwent SRS treatment to cure this disease was used. The data were collected from different medical sources at the Instituto de Radiocirugía del Perú (IRP) between 2005 and 2018 following the process shown in Figure 2.

The variables that were collected from patient data were considered as input data (predictors) and were grouped into 5 categories: sociodemographic (S), clinical (C), treatment (T), angioarchitecture (A), and radiosurgery (R). The variable for patients being cured at 36 months after radiosurgery was considered as output data (response). Table 1 shows the structure of the dataset used in this study.

The dataset is tabular and is made up of 202 records (rows) and 45 variables (columns), in which the rows correspond to the patient data and the columns represent the variables considered in the study. The first 44 variables were considered as input variables to the system (independent variables) and the last column as the output variable (dependent variable), representing patients being cured (cured = 1) or not (cured = 0).

### 2.2. Data Preprocessing

Before carrying out any data processing and because this was a medical application, it was advisable to analyze the data regarding possible confounding variables that could have an undesired impact on our prediction results [23]; for this, we analyzed the possible confounding variables of gender and age.

**Figure 3:**
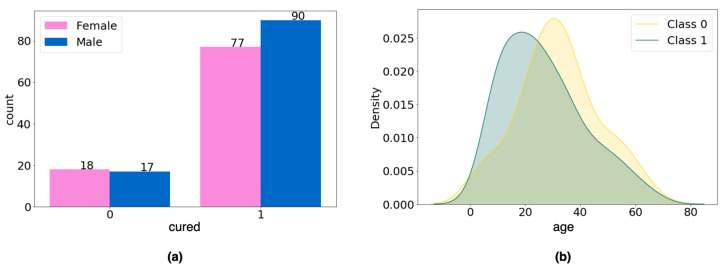
Analysis of variables in the dataset: (**a**) gender variable; (**b**) age variable.

For the categorical variable gender, the chi-square test of homogeneity was performed to verify whether the difference in the number of men and women in each data group was statistically significant, and no difference was found (*p*-value = 0.566; Figure 3a). For the age variable, Student’s *t*-test was applied to verify whether there was a statistically significant difference in age between groups (class 0, mean = 31.97; class 1, mean = 26.72), and again no difference was found (*p*-value = 0.058; Figure 3b).

From this analysis, we concluded that the variables age and gender should not be considered as confounding variables, so we moved forward with the data preprocessing.

Finally, in order to avoid prediction biases and build the ML system effectively, variable selection and data balancing were carried out.

#### 2.2.1. Variable Selection

After an analysis by expert judgment, 6 independent variables were identified that were considered not to influence the prognosis of being cured, so they were excluded from the study (residence, education_level, health_insurance, mri_examination, ct_examination, and das_examination).

Additionally, correlation analysis of the 38 remaining independent variables was carried out; Cramer’s test [24] was applied to identify the linear correlation between categorical variables and Pearson’s test (Pearson’s correlation coefficient) for the numerical variables; in both cases, a threshold value greater than or equal to 0.7 was used to determine the high positive (negative) correlation [25], and 6 correlated variables that exceeded the threshold were identified and discarded from the study (Table 2). The dython library [26], which is available for the Python programming language, was used to perform the calculations. Finally, 12 independent variables were discarded, leaving a dataset made up of 32 independent variables and 1 dependent variable, which were used in the ML system proposed in this study (Table 3).

The final dataset was made up of 202 records, with 32 independent variables and 1 dependent variable, which was divided into two datasets, 75% (*n* = 151) for ML model training and validation and 25% (*n* = 51) for testing. In addition, the 32 independent variables of the training and validation set were normalized using the min–max technique [27].

#### 2.2.2. Data Balancing

**Figure 4:**
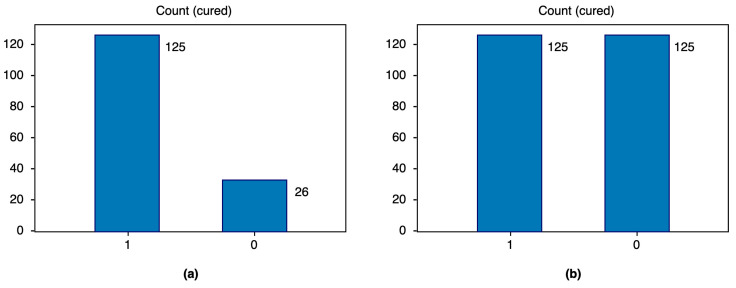
Number of records for each class: (**a**) before and (**b**) after data balancing.

The original training dataset had a data imbalance with respect to the dependent variable, cured, in that it consisted of 125 records of class 1 and 26 of class 0. The imbalance was corrected by applying the synthetic minority oversampling technique (SMOTE), which creates new synthetic instances of the minority class instead of repeating them [28,29]. We obtained 250 records in total; 125 records for each class, as shown in Figure 4.

Finally, two training datasets were obtained: an imbalanced training dataset made up of 151 records, and a balanced training dataset made up of 250 records. Both datasets were represented by a data matrix of dimension *n* × 32, in which the observation i can be expressed as o_i_ = [o_0_, o_1_, …, o_32_] ∈ R*^n^*^×32^, where *n* is the number of observations or records in the dataset.

### 2.3. Machine Learning Models

**Figure 5:**
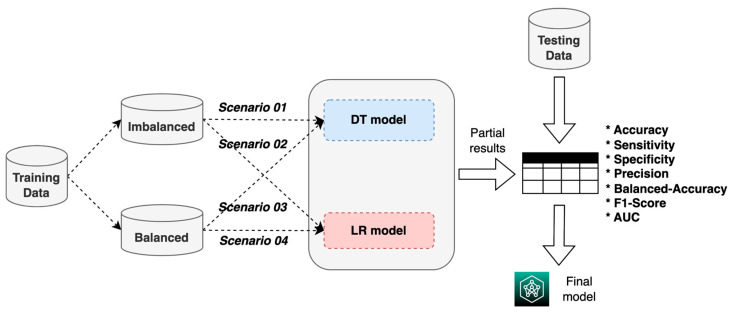
ML model construction, validation, and evaluation process.

For the construction, validation, and evaluation of the ML system, we used the process shown in Figure 5, which consisted of using the two training datasets (balanced and imbalanced) to build and validate the two ML models (DT and LR) in four experimental scenarios; based on the results, the model with the best performance metrics was chosen. Scenario 1 refers to the imbalanced training data with the DT model, scenario 2 refers to the imbalanced training data with the RL model, scenario 3 refers to the balanced training data with the DT model, and scenario 4 refers to the balanced training data with the RL model. The final model’s performance was evaluated by using both the accuracy and the AUC metrics to compare our study’s results with the ones obtained by Oermann et al. [20] and Meng et al. [30]. The accuracy was used to evaluate how well the model predicts the correct label (cured patients) for a given data point, so the ML model can be effectively used in the medical field.

Additionally, the LR method was used to identify the most important factors that determine the probability of patients being cured (clinical interpretability).

In the training phase, the grid search technique [31] was used to find the optimal hyperparameters of the ML models in each of the four scenarios. The set of search values defined for the hyperparameters is given in Table 4.

**Figure 6:**
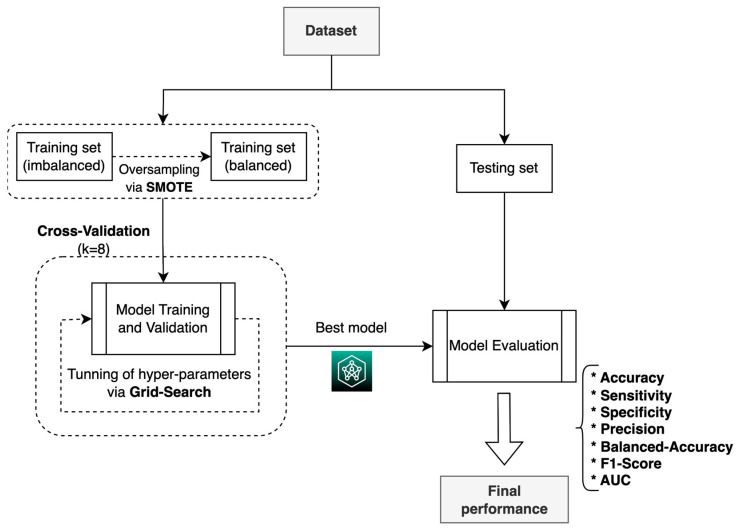
Process used to train the ML models for each of the four scenarios.

During the training process, the resampling technique was used (Figure 6), in which the training dataset was divided into 8 subsets, with 1 set taken for validation and 7 for training, following 8-fold cross-validation, which is a commonly used method for selecting ML models [32,33].

To build the ML models, the scikit-learn 1.0.2 library [34] of Python version 3.8.16 was used in the Google Colab environment. The algorithms and resources built for this research can be found at https://github.com/mirkorodriguez/ml-prediction-mav accessed on 14 December 2023.

## 3. Results

The composition of the study population, the performance of the prediction models, and the explainability of the prediction are presented below.

### 3.1. Study Population

This study included 202 patients with AVM who underwent stereotactic radiosurgery between 2005 and 2018 at the IRP. As shown in Supplementary Figure S1, 167 patients (82.20%) were cured 36 months after the surgical intervention.

Supplementary Table S1 shows the sociodemographic characteristics of the population included this study: 52.97% were men and 47.03% were women; 70.49% of patients were in the age range of 18 to 59 years; 80.69% were from Lima or Callao; 18.82% had a preschool or grade school education and 52.97% had only a high school education; and 42.08% had insurance through the Ministry of Health of Peru (SIS). 

Supplementary Table S2 shows the clinical characteristics of the patients. The average time from radiosurgery to AVM cure (obliteration) was 22.07 months, the average radiation dose was 17.86 Gray, the average AVM diameter was 2.14 cm, and the average number of isocenters applied was 1.35. On average, radiosurgery was performed in a single session.

Supplementary Table S3 shows the statistics of the patients’ previous treatments before SRS. Of the 202 patients, 31 had undergone surgical treatment and 49 had prior embolization. As part of the treatment, 22 only underwent surgery, 40 only embolization, and 9 both surgery and embolization. The embolizing agents were Onyx (52%) and Histoacryl (48%). In total, 155 patients had previous cerebral hemorrhage, 76 developed encephalomalacia, 178 had headache, and 112 had seizures; furthermore, 55% presented some type of deficit (motor, sensory, or cognitive). Regarding the angioarchitecture (characteristics) of the AVM, most (100) were located on the left side of the brain and most (96) were categorized as deep; most treated AVMs (95) had moderately intense flow.

Finally, Supplementary Table S4 shows the anatomical locations of the AVMs, which were mainly found in the basal ganglia (16.83%), frontal lobe (9.9%), insular cortex (6.93%), parieto–occipital region (6.93%), mesio-temporal region (6.93%), and cerebellum (6.44%).

### 3.2. Performance of Prediction Models

The results obtained by the models using the data in the testing set are described below.

Table 5 shows the optimal hyperparameters identified for each scenario that were used in the models for prediction.

**Figure 7:**
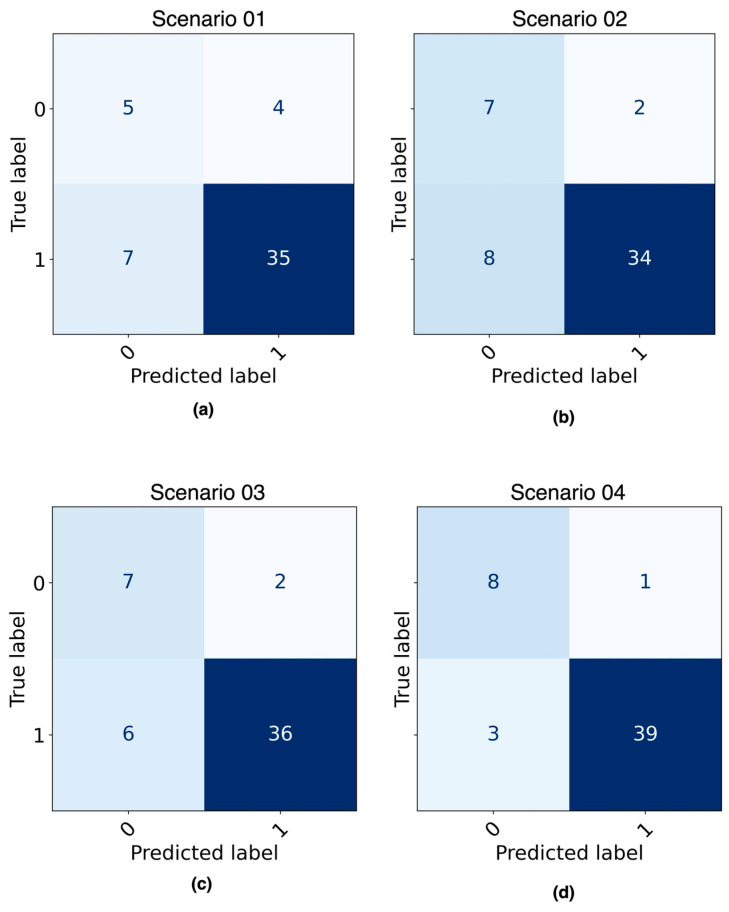
Confusion matrix of ML models evaluated with testing dataset: (**a**) DT model built with imbalanced data; (**b**) DT model built with balanced data; (**c**) LR model built with imbalanced data; (**d**) LR model built with balanced data. The shade of the color represents the quantity of the observations (patients). The bigger the number, the darker the background.

**Figure 8:**
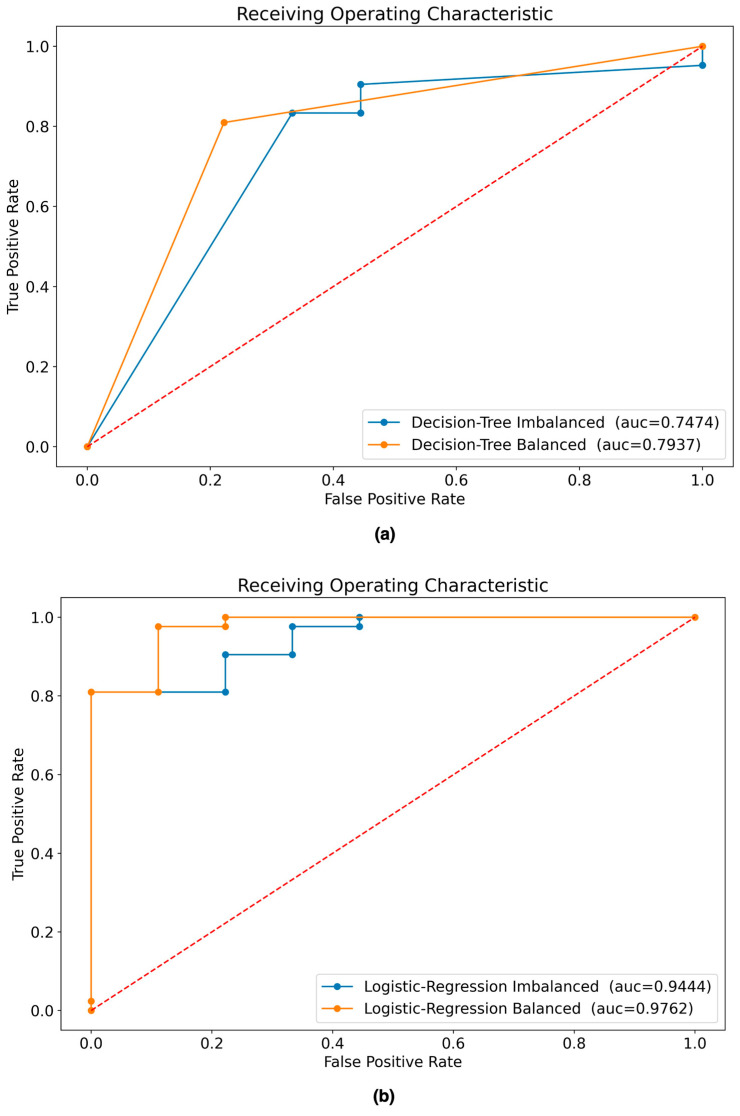
ROC curves of ML models evaluated with testing dataset: (**a**) AUC of DT models built with balanced and imbalanced data; (**b**) AUC of LR models built with balanced and imbalanced data. The dashed line represents a non-discriminatory test.

Figure 7 shows the confusion matrices obtained as a result of evaluating the best ML model from each of the four predefined scenarios with the testing dataset. Figure 8 shows the AUC curve for each scenario.

Table 6 shows the results of the experiments with the four scenarios in terms of their performance metrics for both the training and testing datasets. The best model according to the performance metrics in the testing dataset is the LR model built with the balanced dataset.

### 3.3. Explainability of Models

In order to gain a general idea about the explainability of the results obtained by the models used in this research, the LR model built with balanced data (scenario 4) was used based on its good prediction results and its interpretability through the calculation of the odds ratio (importance) [35]. Table 7 shows the variables (features) and their level of importance in explaining the probability of patients with AVM being cured 36 months after SRS, among which 18 have a negative influence and 14 have a positive influence. The five most important variables that positively influence being cured are (1) the location of the AVM (side_avm), (2) the occupation of the patient (occupation), (3) the presence of bleeding in the AVM (hemorrhage), (4) previous cranial surgery (prev_cran_surgery), and (5) the type of venous drainage (type_venous_drainage). It is important to highlight that the patient’s occupation is an antecedent of the disease, but it is not clinically relevant; however, it is an interesting finding that should be evaluated in greater detail in another study. 

## 4. Discussion

Inspired by the use of ML techniques in medicine [36,37,38,39,40,41,42], and specifically for the prognosis of patients with AVM [19,20,30], this study proposed a method that makes it possible to predict whether or not a patient with AVM who undergoes SRS will be cured at 36 months after the intervention. We found that using ML techniques for the prognosis of patients with AVMs is possible. Our approach involved evaluating four scenarios using two ML models and two datasets (imbalanced and balanced data). After following a standard process to build the ML models, in which oversampling, grid search, and cross-validation techniques were also applied, it was found that the best model to predict whether patients with AVM would be cured is the LR model trained with balanced data (accuracy 0.92, AUC 0.98). The LR model was superior to the DT model even when trained with imbalanced data, as shown in Table 6. The data preprocessing (selection of variables and balancing) performed in this study led to significantly higher results for the two models (DT and LR) than when the data were not preprocessed, so we can argue that data preprocessing should be included in any approach that uses an ML model. In addition, the results obtained in this study (accuracy 0.92 and AUC 0.98) were found to be superior to the results obtained in other studies using similar procedures, such as those by Oermann et al. [20] and Meng et al. [30], who obtained an accuracy of 0.74 and 0.83, and an AUC of 0.71 and 0.77, respectively.

From the clinical perspective, it is observed that the data used in this study have acceptable homogeneity for the radiosurgery protocol: AVM diameter of 2.14 cm (SD = 0.89), applied radiation dose of 17.86 Gy (SD = 4.44), and number of isocenters of 1.35 (SD = 0.56); all of this, together with other technical and morphological factors, allowed for the effective application of ML techniques to individualize the AVMs that will respond positively to radiosurgery treatment. The LR model is the one that best predicts the SRS outcomes and the variables that positively influence determining whether a patient will be cured are (1) the location in the basal ganglia, which coincides with previous studies [43]; additionally, the location of the AVM on the left side of the brain as an important factor is due to the fact that the sample is not completely random; (2) deep venous drainage, which occurs at the level of the basal ganglia or midbrain is considered not treatable with other techniques due to the high risk involved; (3) the occupational group, which denotes a population of children and adolescents who tend to have a good response to radiosurgery, was expected and also coincides with results from other studies [44]. In addition, it is important to highlight that both the history of bleeding in the AVM and the presence of previous surgical treatment are key prognostic factors, as it is shown in our study, where 71 (35.14%) of the patients had previous treatment either through conventional neurosurgery, embolization, or both, which contributed to improving the favorable prognosis of AVMs by reducing their size or altering the hemodynamics of the residual AVM, which ultimately favors its healing.

The importance of the results of this study goes beyond the possibility of using this method for the medical prognosis of patients with AVM; it also allows us to confirm that it is possible to use an ML model, understood as a generalizable framework, in medicine, by using historical data to predict the future. We believe that the ML algorithms that process clinical and imaging data in a personalized way can effectively help in decision-making to predict which patients with cerebral AVM could benefit from being cured by treatment with stereotactic radiosurgery. In this case, we used historical information over a 14-year time horizon, from which sociodemographic and medical data were collected to build an ML system that achieved very good prediction results and could be used as a tool by medical professionals for decision-making when dealing with new AVM cases.

Finally, the proposed approach for the prognosis and explainability of whether patients with AVM will be cured has no limitations; however, the results of these models are limited to the dataset used in this study, so its application in medical practice requires more experiments with larger amounts of data and the possibility of including additional medical variables should also be evaluated. Also, it is important to remark that the two ML models used in this study are considered transparent models, or “white box” models [21], the results of which are easy to interpret; however, it would be important to contrast the interpretability with more sophisticated explainability techniques such as local interpretable model-agnostic explanations (LIME), Shapley additive explanations (SHAP), and others, which are focused on identifying the most important predictors for any type of ML model, including those considered “black box” models.

## Figures and Tables

**Table 1:** Dataset structure.

Cat.	Variable Name	Description	Values
(S)	gender	Sexual/gender identity	1 = male; 0 = female
(S)	age	Chronological age	4–75
(S)	residence	Place of residence (city where patient lived during treatment)	1 = Lima or Callao, Peru; 2 = outside Lima or Callao in Peru; 3 = outside Peru
(S)	occupation	Principal work or business	1 = professional with bachelor’s or technical degree; 2 = general worker; 3 = housewife; 4 = police officer or similar; 5 = undergraduate student; 6 = school student; 7 = unemployed; 8 = self-employed
(S)	education_level	Level of education	1 = preschool; 2 = primary school; 3 = secondary school; 4 = higher education
(S)	health_insurance	Type of health insurance	1 = private; 2 = EsSalud; 3 = SIS; 4 = personal; 5 = military or similar
(C)	hemorrhage	Presence of bleeding on a computerized tomography (CT) scan in brain AVM before radiosurgery	1 = yes; 0 = no
(C)	hemorrhage_type	Type of bleeding in brain AVM	1 = parenchymal; 2 = ventricular; 3 = parenchymal and ventricular; 4 = no hemorrhage present
(C)	headache	Persistent headache before radiosurgery	1 = yes; 0 = no
(C)	seizures	Presence of seizures at time of diagnosis	1 = yes; 0 = no
(C)	encephalomalacia	Localized softening of brain substance due to bleeding or inflammation before radiosurgery	1 = yes; 0 = no
(C)	other_diseases	Presence of other systemic or degenerative diseases	1 = yes; 0 = no
(C)	deficit	Type of deficit in patient’s senses before radiosurgery	1 = motor deficit; 2 = sensory deficit; 3 = cognitive deficit; 4 = no deficit observed
(C)	karnofsky_scale	Measurement for classification of functional impairment	0–100%
(C)	glasgow_coma_scale	Assessment of impaired consciousness in response to defined stimuli	3–15
(C)	spetzler_martin_scale	Estimation of risk of open neurosurgery for patients with brain AVM, by evaluating AVM size, pattern of venous drainage, and eloquence of brain location	0–5
(C)	buffalo_scale	Grading system for endovascular treatment of brain AVMs	
(C)	virginia_scale	Scale to predict favorable outcomes for brain AVM patients treated with gamma knife radiosurgery	0–4
(T)	prev_cran_surgery	Previous open cranial surgery	1 = yes; 0 = no
(T)	embolization	Embolization procedure to occlude brain AVM before radiosurgery	1 = yes; 0 = no
(T)	embolization_agent	Type of material used for embolization procedure	1 = Onyx; 2 = Histoacryl; 3 = none
(T)	prev_surgery_or_embolization	Surgery or embolization before radiosurgery procedure	1 = surgery; 2 = embolization; 3 = surgery and embolization; 4 = none
(A)	localization_avm	Anatomical location of brain AVM	1 = frontal lobe; 2 = temporal lobe; 3 = parietal lobe; 4 = occipital lobe; 5 = cerebral corpus callosum; 6 = insular cortex; 7 = basal ganglia; 8 = cerebellum; 9 = ventricular; 10 = vermis; 11 = frontomesial; 12 = frontoparietal; 13 = frontotemporal; 14 = mesencephalon; 15 = mesio-occipital; 16 = mesio-parietal; 17 = parieto-occipital; 18 = protuberance; 19 = mesio-temporal; 20 = temporo-occipital; 21 = temporo-parietal; 22 = brainstem
(A)	venous_aneurysm	Presence of venous aneurysm along with brain AVM	1 = yes; 0 = no
(A)	arterial_aneurysm	Presence of arterial aneurysm along with brain AVM	1 = yes; 0 = no
(A)	dolichoectasia	Elongation, dilatation, and distension of brain AVM drainage veins	1 = yes; 0 = no
(A)	num_afferent_vessels	Number of arteries feeding brain AVM	Number
(A)	depth_avm	Depth of brain AVM inside cranial structure	1 = cortical; 2 = subcortical; 3 = cortico-subcortical; 4 = deep; 5 = ventricular
(A)	diameter_avm	Largest diameter of brain AVM in centimeters	0.5–8.0 cm
(A)	side_avm	Brain side where AVM is located	1 = right; 2 = left; 3 = middle
(A)	expansion_shape_avm	Shape of AVM expansion in cerebral area	1 = compact; 2 = fuzzy; 3 = scattered mixed
(A)	type_venous_drainage	Drainage type of venous blood in brain AVM	1 = superficial; 2 = deep; 3 = mixed
(A)	eloquence	Brain AVM is in a zone that compromises vital functions	1 = yes; 0 = no
(A)	type_circulation_drainage	Type of circulation of drainage in brain AVM	1 = superficial venous; 2 = deep venous
(A)	blood_flow_velocity	Blood flow velocity in brain AVM	1 = slow; 2 = moderate; 3 = fast
(A)	venous_stenosis	Narrowing of venous vessel lumen at outlet of drainage of brain AVM	1 = yes; 0 = no
(A)	volume_avm	Volume of brain AVM mass in cubic centimeters	0.05–75 cc
(A)	num_radiosurgeries	Number of radiosurgeries needed to stabilize brain AVM	Number
(A)	mri_examination	Brain AVM was examined by magnetic resonance imaging (MRI)	1 = yes; 0 = no
(A)	ct_examination	Brain AVM was examined by CT	1 = yes; 0 = no
(A)	das_examination	Brain AVM was examined by digital angiography system (DAS)	1 = yes; 0 = no
(R)	num_isocenters	Number of iso-centers to cover and treat brain AVM	Number
(R)	radiation_doses	Dose of radiation applied to brain AVM during radiosurgery in Gray units	1–50 Gy
(R)	isodosis	Percentage of isodosis applied during radiosurgery of brain AVM	40–80%
(R)	cured	Brain AVM is cured within 3 years of radiosurgery, as indicated by cerebral angiography	1 = patient was cured; 0 = patient was not cured

S, sociodemographic; C, clinical; T, treatment; A, angioarchitecture; R, radiosurgery.

**Table 2:** Variables discarded from the study.

Discarded Variables	Method	Threshold
residence, education_level, health_insurance, mri_examination, ct_examination, das_examination	Expert judgment	n.a.
hemorrhage_type, embolization_agent, prev_surgery_or_embolization, spetzler_martin_scale, type_circulation_drainage	Cramer’s V test	0.7
diameter_avm	Pearson’s test	0.7

**Table 3:** Variables selected for the study.

Id	Variable Name	Id	Variable Name	Id	Variable Name
1	gender	12	buffalo_scale	23	expansion_shape_avm
2	age	13	virginia_scale	24	type_venous_drainage
3	occupation	14	prev_cran_surgery	25	eloquence
4	hemorrhage	15	embolization	26	blood_flow_velocity
5	headache	16	localization_avm	27	venous_stenosis
6	seizures	17	venous_aneurysm	28	volume_avm
7	encephalomalacia	18	arterial_aneurysm	29	num_radiosurgeries
8	other_diseases	19	dolichoectasia	30	num_isocenters
9	deficit	20	num_afferent_vessels	31	radiation_doses
10	karnofsky_scale	21	depth_avm	32	isodosis
11	glasgow_coma_scale	22	side_avm	33	cured *

* Dependent variable.

**Table 4:** Search space for tuning hyperparameter values.

Model	Parameters	Grid Search Space
Decision tree (DT)	max_depth	2–9
	criterion	gini, entropy
Logistic regression (LR)	penalty	l1, l2
	solver	liblinear
	C	0.001, 0.01, 0.1, 1, 10, 100, 100
	max_iter	1000, 5000

**Table 5:** Calibrated hyperparameters for each model found during the training process.

Scenario	Dataset + Model	Parameters	Value
01	Imbalanced + DT	max_depth	4
		criterion	gini
02	Balanced + DT	max_depth	9
		criterion	entropy
03	Imbalanced + LR	penalty	l1
		solver	liblinear
		C	10
		max_iter	1000
04	Balanced + LR	penalty	l1
		solver	liblinear
		C	10
		max_iter	1000

**Table 6:** Summary of models’ performance.

Dataset	Model Name	Accuracy	Sensitivity	Specificity	Precision	Bal. Accuracy	F1-Score	AUC
Training	DT *	0.92	0.93	0.88	0.97	0.91	0.95	0.94
	DT (imbalanced)	0.94	0.96	0.85	0.97	0.90	0.96	0.92
	DT (balanced)	0.98	0.98	0.99	0.99	0.98	0.98	0.98
	LR *	0.95	0.96	0.92	0.98	0.94	0.97	0.96
	LR (imbalanced)	0.97	0.96	1.00	1.00	0.98	0.98	0.99
	LR (balanced)	0.96	0.97	0.96	0.96	0.96	0.96	0.99
Testing	DT *	0.76	0.86	0.33	0.86	0.60	0.86	0.62
	DT (imbalanced)	0.78	0.83	0.56	0.90	0.69	0.86	0.75
	DT (balanced)	0.80	0.81	0.78	0.94	0.79	0.87	0.79
	LR *	0.80	0.81	0.78	0.94	0.79	0.87	0.93
	LR (imbalanced)	0.84	0.86	0.78	0.95	0.82	0.90	0.94
	**LR (balanced)**	**0.92**	**0.93**	**0.89**	**0.98**	**0.91**	**0.95**	**0.98**

* Models built without any data preprocessing used as a baseline for comparison.

**Table 7:** Importance of variables in LR model calculated via odds ratio.

Feature	Coef.	Importance
side_avm	3.69	3.99 × 10^1^
occupation	3.68	3.98 × 10^1^
hemorrhage	3.61	3.71 × 10^1^
prev_cran_surgery	2.81	1.66 × 10^1^
type_venous_drainage	2.12	8.35 × 10^0^
deficit	1.01	2.74 × 10^0^
eloquence	0.98	2.66 × 10^0^
gender	0.48	1.61 × 10^0^
seizures	0.43	1.53 × 10^0^
karnofsky_scale	0.00	1.00 × 10^0^
virginia_scale	0.00	1.00 × 10^0^
num_isocenters	0.00	1.00 × 10^0^
num_radiosurgeries	0.00	1.00 × 10^0^
arterial_aneurysm	0.00	1.00 × 10^0^
headache	−0.01	9.90 × 10^−1^
glasgow_coma_scale	−0.06	9.39 × 10^−1^
buffalo_scale	−0.59	5.52 × 10^−1^
venous_stenosis	−1.08	3.39 × 10^−1^
radiation_doses	−1.11	3.28 × 10^−1^
num_afferent_vessels	−1.23	2.93 × 10^−1^
other_diseases	−1.29	2.74 × 10^−1^
venous_aneurysm	−1.34	2.61 × 10^−1^
age	−2.23	1.07 × 10^−1^
encephalomalacia	−3.07	4.64 × 10^−2^
localization_avm	−3.11	4.47 × 10^−2^
depth_avm	−3.23	3.95 × 10^−2^
expansion_shape_avm	−4.01	1.81 × 10^−2^
isodosis	−4.73	8.82 × 10^−3^
embolization	−4.74	8.75 × 10^−3^
dolichoectasia	−5.67	3.46 × 10^−3^
blood_flow_velocity	−7.41	6.07 × 10^−4^
volume_avm	−21.47	4.75 × 10^−10^

## Data Availability

The data presented in this study are available on request from the corresponding author. The data are not publicly available due to institutional (Instituto de Radiocirugía del Perú) regulations and the protection of patient privacy.

## Supplementary Materials

The following supporting information can be downloaded at https://www.mdpi.com/article/10.3390/diagnostics14010022/s1, Figure S1: Distribution of cured variable; Table S1: Sociodemographic characteristics of study population; Table S2: Clinical and radiosurgery characteristics of study population; Table S3: Angioarchitecture and treatment characteristics of study population; Table S4: Locations of AVM in study population.
