# Supplementary material for: Machine Learning Approach for Analyzing 3-Year Outcomes of Patients with Brain Arteriovenous Malformation (AVM) after Stereotactic Radiosurgery (SRS)
Source: Diagnostics (Basel). 2023 Dec 22;14(1):22. doi: 10.3390/diagnostics14010022 (PMC10871108; doi:10.3390/diagnostics14010022)
Supplement: Supplementary file 1 [file diagnostics-14-00022-s001.zip › diagnostics-2771639-supplementary.pdf]

Supplementary Material:

Machine learning approach for analyzing 3-year outcomes of patients with brain arteriovenous malformation (AVM) after stereotactic radiosurgery (SRS)

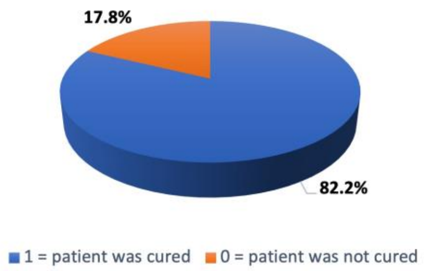

Figure S1. Distribution of cured variable.

Table S1. Sociodemographic characteristics of study population.

Variable	Value	Number	%
Gender	Male	107	52.97
	Female	95	47.03
Age	Child (1–11)	30	14.85
	Teenager (12–17)	24	11.88
	Young boy (18–29)	66	32.37
	Adult (30–59)	77	38.12
	Older adult (60+)	5	2.48
Residence	Lima or Callao in Perú	163	80.69
	Outside Lima or Callao in Perú	37	18.32
	Outside Perú	2	0.99
Occupation	Professional with bachelor's or technical degree	35	17.33
	General worker	24	11.88
	Housewife	34	16.83
	Police officer or similar	9	4.46
	Undergraduate student	20	9.90
	School student	56	27.72
	Unemployed	10	4.95
	Self-employed	14	6.93
Education level	Preschool	5	2.48
	Primary school	33	16.34
	Secondary school	107	52.97
	Higher education	57	28.22
Health insurance	Private	20	9.90
	EsSalud	29	14.36
	SIS	85	42.08
	Personal	49	24.26
	Military or similar	19	9.41
	**Total**	**202**	**100.0**

Table S2. Clinical and radiosurgery characteristics of study population.

Variable	Mean	Std Dev	Min	Median	Max
Age	27.63	14.90	4	25	68
karnofsky_scale	82.78	9.37	40	80	100
glasgow_coma_scale	14.80	0.46	12	15	15
spetzler_martin_scale	2.55	0.89	1	3	5
buffalo_scale	2.43	0.98	0	2	5
virginia_scale	2.08	0.82	0	2	4
diameter_avm	2.14	0.89	0.5	2.1	6
volume_avm	6.30	8.33	0.063	4	75
num_afferent_vessels	2.51	0.92	1	2	6
num_radiosurgeries	1.36	1.56	1	1	10
num_isocenters	1.35	0.56	1	1	4
radiation_doses	17.86	4.44	10	17	40
isodosis	69.31	14.37	50	80	90
cured	22.07	6.47	6	24	36

Table S3. Angioarchitecture and treatment characteristics of study population.

Variable	Frequency by category	Values
prev_cran_surgery	1: 31; 2: 171	1 = yes; 0 = no
embolization	1: 49; 2: 153	1 = yes; 0 = no
embolization_agent	1: 26; 2: 24; 3: 152	1 = Onyx; 2 = Histoacryl; 3 = none
prev_surgery_or_embolization	1: 22; 2: 40; 3: 9; 4: 131	1 = surgery; 2 = embolization; 3 = surgery and embolization; 4 = none
hemorrhage	1: 155; 2: 47	1 = yes; 2 = no
hemorrhage_type	1: 91; 2: 13; 3: 29; 4: 69	1 = parenchymal; 2 = ventricular; 3 = parenchymal and ventricular; 4 = none
headache	1: 178; 2: 24	1 = yes; 0 = no
seizures	1: 112; 2: 90	1 = yes; 0 = no
encephalomalacia	1: 76; 2: 126	1 = yes; 0 = no
deficit	1: 53; 2: 26; 3: 32; 4: 91	1 = motor deficit; 2 = sensory deficit; 3 = cognitive deficit; 4 = no deficit observed
venous_aneurysm	1: 58; 2: 144	1 = yes; 0 = no
arterial_aneurysm	1: 3; 2: 199	1 = yes; 0 = no
dolichoectasia	1: 140; 2: 62	1 = yes; 0 = no
depth_avm	1: 9; 2: 48; 3: 47; 4: 96; 5: 2	1 = cortical; 2 = subcortical; 3 = cortico-subcortical; 4 = deep; 5 = ventricular
side_avm	1: 85; 2: 100; 3: 17	1 = right; 2 = left; 3 = middle
expansion_shape_avm	1: 145; 2: 39; 3: 18	1 = compact; 2 = fuzzy; 3 = scattered mixed
type_venous_drainage	1: 75; 2: 114; 3: 13	1 = superficial; 2 = deep; 3 = mixed
eloquence	1: 63; 2: 139	1 = yes; 0 = no
blood_flow_velocity	1: 80; 2: 95; 3: 27	1 = slow; 2 = moderate; 3 = fast
venous_stenosis	1: 52; 2: 150	1 = yes; 0 = no

Table S4. Locations of AVM in study population.

AVM location	Number	%
Frontal lobe	20	9.90
Temporal lobe	11	5.45
Parietal lobe	11	5.45
Occipital lobe	7	3.47
Cerebral corpus callosum	12	5.94
Insular cortex	14	6.93
Basal ganglia	34	16.83
Cerebellum	13	6.44
Ventricular	10	4.95
Vermis	3	1.49
Frontomesial	6	2.97
Frontoparietal	9	4.46
Frontotemporal	3	1.49
Mesencephalon	7	3.47
Mesio-occipital	3	1.49
Mesio-parietal	2	0.99
Parieto-occipital	14	6.93
Protuberance	3	1.49
Mesio-temporal	14	6.93
Temporo-occipital	3	1.49
Temporo-parietal	1	0.49
Brainstem	2	0.99
Total	202	100.0
